# Dietary Bioactive Peptide Alanyl-Glutamine Attenuates Dextran Sodium Sulfate-Induced Colitis by Modulating Gut Microbiota

**DOI:** 10.1155/2021/5543003

**Published:** 2021-05-08

**Authors:** Qingbiao Xu, Mingyang Hu, Min Li, Jinxiu Hou, Xianghua Zhang, Ya Gao, Bahram Chachar, Xiang Li

**Affiliations:** ^1^College of Animal Sciences and Technology, Huazhong Agricultural University, Wuhan 430070, China; ^2^Department of Animal Nutrition, Faculty of Veterinary and Animal Sciences, Lasbela University of Agriculture, Water and Marine Sciences, Uthal, Balochistan 90150, Pakistan

## Abstract

Inflammatory bowel disease (IBD) is a chronic intestinal disorder threatening human health. Di-peptide alanyl-glutamine (Ala-Gln) has various beneficial effects on gut health. However, its role and functional mechanism in treating IBD are still not clear. Therefore, the protective effects of Ala-Gln and glutamine (Gln) on dextran sulfate sodium- (DSS-) induced colitic mice were investigated in this study. The results showed that oral supplementation of Ala-Gln or Gln significantly attenuated the colitis symptoms in mice, including body weight loss, colon length, disease activity index, histological scores, and tissue apoptosis. The concentrations of interleukin- (IL-) 1*β*, IL-6, tumor necrosis factor-*α*, and myeloperoxidase were significantly decreased, while the concentrations of immunoglobulins (IgA, IgG, and IgM) and superoxide dismutase were significantly increased by Ala-Gln or Gln supplementation. The expression of occludin and peptide transporter 1 (PepT1) was significantly increased by Ala-Gln or Gln. Interestingly, Ala-Gln had better beneficial effects than Gln in alleviating colitis. In addition, 16S rDNA sequencing showed that the DSS-induced shifts of the microbiome (community diversity, evenness, richness, and composition) in the mouse colon were restored by Gln and Ala-Gln, including *Lactobacillus*, *Bacteroides_acidifaciens*, *Bacteroidales*, *Firmicutes*, *Clostridia*, *Helicobacter*, and *Bacteroides*. Correspondingly, the functions of the microflora metabolism pathways were also rescued by Ala-Gln, including fatty acid metabolism, membrane transporters, infectious diseases, and immune system. In conclusion, the results revealed that Ala-Gln can prevent colitis through PepT1, enhancing the intestinal barrier and modulating gut microbiota and microflora metabolites.

## 1. Introduction

Inflammatory bowel disease (IBD, including ulcerative colitis) is a chronic gastrointestinal disorder caused by inflammation or oxidative stress in the colon, which has threatened the human health and has various colitis symptoms, such as gut bleeding, bloody diarrhea, body weight (BW) loss, epithelial cell loss, neutrophil infiltration, and the release of proinflammatory mediators (e.g., interleukin- (IL-) 1*β*, IL-4, IL-5, IL-6, IL-8, and tumor necrosis factor- (TNF-) *α*) [1]. However, the use of drugs to treat IBDs often has side effects. The application of bioactive peptides with the potential to manage chronic intestinal inflammation comes into people's view [1].

Glutamine (Gln) is a well-studied amino acid with immune-modulating effects [2, 3], and it can alleviate intestinal inflammation [4]. The Gln can be utilized to maintain the intestinal structure and function [5], and it also can mediate protein synthesis by intestinal microorganisms [6]. Gln used in human and animal studies is often in the form of alanyl-glutamine (Ala-Gln) [3]. However, the difference of the effects between Ala-Gln and Gln is still not clear. Intriguingly, Ala-Gln has a superior preventive effect on intestinal damage with more mucosal weight, protein content, and villus height than those in mice with alanine plus Gln mixture [7]. Ala-Gln had a better effect on improving enterocyte proliferation than Gly-Gln or Gln [8]. In other words, Ala-Gln may serve as a bioactive peptide and has better effects than Gln or alanine alone.

It was reported that Ala-Gln can alleviate inflammation via inhibiting cytokine expression and regulating T cells in colitic mice [3, 9, 10] and alleviates the allergic inflammation of the airways and lungs through modulating gut microbiota and their metabolites in mice [11]. The Ala-Gln can attenuate metabolic stress, enhance immunity [12], and inhibit intestinal mucositis in mice [13]. In addition, Ala-Gln can also improve the gastrointestinal epithelial structure and immune status of early-weaned calves [14]. In a number of studies, dextran sulfate sodium- (DSS-) induced mouse colitis is a widely used model to study IBD with the features of ulcerative colitis [1, 15]. The peptide Ala-Gln has attracted an increasing interest of scientists. However, the role and functional mechanism of Ala-Gln on colitis are still unclear, particularly in the gut microbiota. Therefore, the objective of this study is to explore the effects of Ala-Gln on DSS-induced acute colitis in mice.

## 2. Materials and Methods

### 2.1. Mouse Treatment

Forty male mice (6-7 weeks old; Institute of Cancer Research; body weight (BW), 28-32 g) were obtained from Liaoning Changsheng Biotechnology Co., Ltd. (Benxi, China). Ala-Gln was obtained from Wuhan Jetide Biotech Co., Ltd. (Wuhan, China). The DSS was purchased from Sangon Biotech Co., Ltd. (A600160-0250, Shanghai, China). Mice were randomly assigned to four groups (*n* = 10): control, DSS, DSS+Gln (Gln), and DSS+Ala-Gln (Ala-Gln). Mice in the Gln or Ala-Gln group received Gln (2.5%, *w*/*v*, 500 mg Gln/kg BW/d) or Ala-Gln (3.75%, *w*/*v*, 750 mg Ala-Gln/kg BW/d, equivalent to 500 mg Gln/kg BW/d) dissolved in sterilized distilled water by daily intragastric gavage from day 1 to 14. From day 8 to 14, the mice in DSS, Gln, and Ala-Gln groups were given 3% DSS (*w*/*v*) in water. Mice were kept under the controlled conditions of 12 h light/dark, constant temperature (25°C), and humidity (50 ± 5%). The amount of Gln and Ala-Gln given to mice is a safe dose used in clinical studies [10, 15].

### 2.2. Disease Activity Index (DAI) and Colon Histologic Analysis

The DAI was assessed with the score sum according to BW loss, stool consistency and bleeding, and mouse condition as previous reports [16, 17]. After sacrificing mice on day 15, the colon length was measured, and the colon tissue (~3.5 cm proximal to the anus) was fixed with 10% buffered formalin and stained with hematoxylin and eosin (HE) to obtain the morphology structure. The tissue damage was evaluated using the degree of inflammatory cell infiltration based on previous descriptions [16, 18]. In addition, the cell apoptotic level was assessed by the terminal deoxynucleotidyl transferase dUTP nick end labeling (TUNEL) assay by using a cell death detection kit with the nuclei stained with DAPI (Roche, Basel, Switzerland). The morphology was observed using fluorescent microscopy (DMIL LED, Leica, Germany).

### 2.3. Enzyme-Linked Immunosorbent Assay (ELISA)

The serum was obtained with blood being centrifuged for 15 min at 1500 g. The concentrations of immunoglobulin (Ig) A, IgG, and IgM in the serum were detected by using ELISA kits (Nanjing Jiancheng Bioengineering Institute, Nanjing, China). The contents of IL-1*β*, IL-6, TNF-*α*, superoxide dismutase (SOD), and myeloperoxidase (MPO) in colon tissues were measured by using ELISA kits according to the manufacturer's instructions [19]. The content of tissue protein was measured using the Bicinchoninic Acid Protein Assay Kit (P0011, Beyotime, Shanghai, China) and analyzed using a microplate reader (Bio-Rad, 680, Hercules, CA, USA).

### 2.4. Western Blot

Colon tissue was lysed using radioimmunoprecipitation assay lysis buffer (P0013B, Beyotime, Shanghai, China) and PMSF (ST506, Beyotime, Shanghai, China), and the lysate was homogenized and centrifuged to obtain the supernatant. The sample in the supernatant with loading buffer (SibEnzyme, Russia) was kept at 95°C to denature the protein. The sample was condensed and separated via the appropriate concentration of SDS gel electrophoresis and then transferred to a PVDF membrane (Sigma-Aldrich, UK), which was then blocked in 5% BSA. After being washed using TBST buffer, the PVDF membrane was incubated with primary antibodies at 4°C overnight (1 : 1000), including *β*-actin (4970S, CST), PepT1 (sc-373742, Santa Cruz, CA, USA), occludin (sc-133255, Santa Cruz, CA, USA), extracellular signal-regulated kinase (ERK; CST 4695S), pERK (CST 4370S), p38 (CST 8690S), pp38 (CST 4511S), c-Jun N-terminal kinase (JNK; CST 3708S), and pJNK (CST 9255S). Subsequently, the membrane was incubated with an HRP-conjugated secondary antibody (1 : 8000, CST, Danvers, MA, USA). The bands were observed by using Chemilmaging software (Baygene Biotech, China) with fluorescence excited using HRP-conjugated ECL Western Blotting Substrate (Tanon, Shanghai, China). Band intensity was measured using ImageJ software (NIH, USA) and normalized using *β*-actin.

### 2.5. Gut Microbiota

The DNA of mouse colon luminal content was extracted by using the QIAamp DNA Stool Mini Kit (Qiagen, Hilden, Germany). The primers targeting V3 to V4 regions of the 16S rDNA sequence were amplified using polymerase chain reaction (PCR). The PCR products were purified using Agencourt AMPure XP and sequenced using a HiSeq platform. The raw data was filtered, and the Operational Taxonomic Units (OTUs; >97% similarity) were clustered by using USEARCH and the data was analyzed by using an RDP classifier (>80% confidence). The diversity indices of alpha and beta were analyzed using Quantitative Insights into Microbial Ecology (QIIME; http://qiime.org/). The distances among samples were computed using principal component analysis (PCA) and partial least squares discriminant analysis (PLS-DA). In addition, linear discriminant analysis (LDA) effect size (LEfSe) was used to determine taxa, which can characterize each population (LDA score > 4) to discover biomarkers. A Venn diagram was drawn by using R software and used to present OTU overlap among samples. Additionally, the cladogram plot was drawn using Figure Tree software to identify corresponding group biomarkers (http://tree.bio.ed.ac.uk/software/figtree/). The taxonomical levels were carried out at the phylum, class, order, genus, and species levels. Moreover, the function of the metagenome was predicted using Phylogenetic Investigation of Communities by Reconstruction of Unobserved States (PICRUSt; http://picrust.github.com) and Kyoto Encyclopedia of Genes and Genomes (KEGG) levels 1, 2, and 3.

### 2.6. Statistical Analysis

Data analysis was performed by Student's *t*-test between two groups and one-way ANOVA followed by Tukey's comparison among multiple groups by using SPSS software (SPSS Inc., Chicago, IL, USA). The results were presented as means ± standard error of mean (SEM). *p* < 0.05 was considered statistically significant.

## 3. Results

### 3.1. Ala-Gln Alleviated DSS-Induced Mouse Colitis

**Figure 1:**
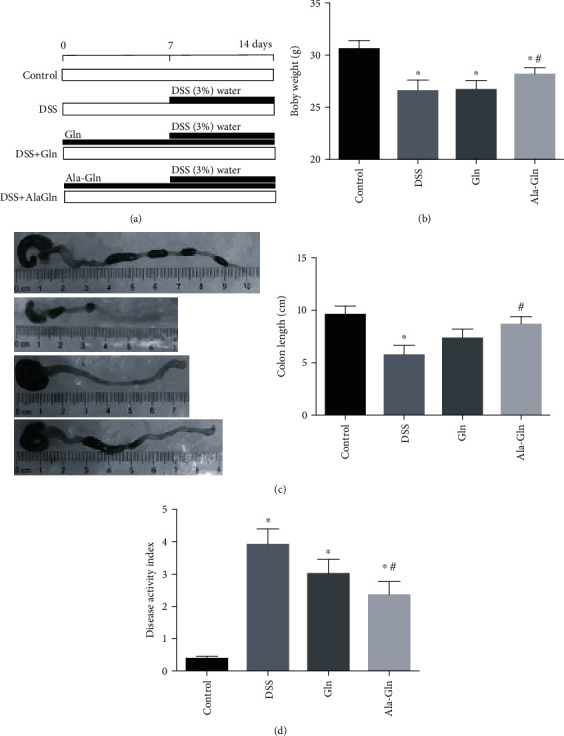
Supplementations of Gln and Ala-Gln attenuated the mouse colitis symptoms induced by DSS. (a) Experimental timeline. (b) Body weight. (c) Colon length. (d) Disease activity index. Results were shown as means ± SEM (*n* = 6). ^∗^*p* < 0.05 versus control group; ^#^*p* < 0.05 versus DSS group.

The experiment was carried out according to a timeline as shown in Figure 1(a). Colitis symptoms had appeared in mice after oral treatment with DSS for a week, including low BW (Figure 1(b)), short colon length (Figure 1(c)), and high DAI (Figure 1(d)). However, the BW and colon length were significantly upregulated by Ala-Gln (*p* < 0.05) (Figures 1(b) and 1(c)). The DSS-induced high DAI was significantly decreased by Ala-Gln (*p* < 0.05) (Figure 1(d)). However, the treatment of Gln did not significantly alleviate DSS-induced mouse colitis. These results suggested that the DSS-induced colitis symptom was dramatically alleviated by Ala-Gln.

### 3.2. Colon Tissue Damage and Apoptosis

**Figure 2:**
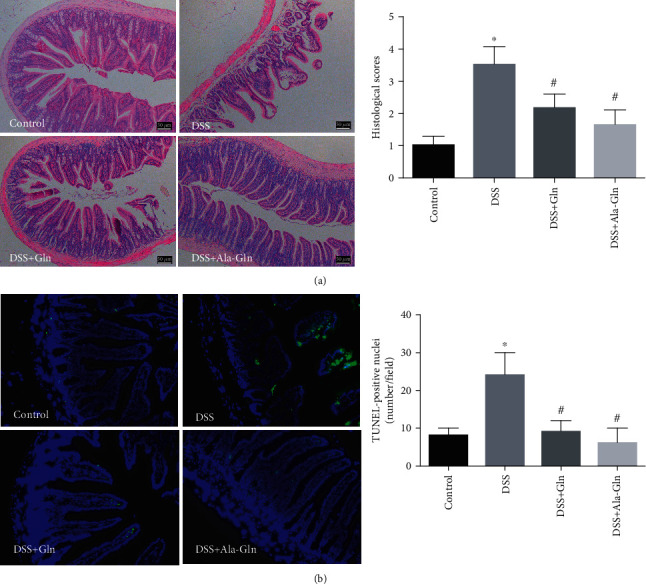
Supplementations of Gln and Ala-Gln rescued DSS-induced mouse colon histological damage. (a) The HE staining micrographs and histological scoring of mouse colon morphology. Bar = 50 *μ*m. (b) Fluorescent micrographs and quantitation of TUNEL (green) and DAPI (blue) of colon tissue. Bar = 100 *μ*m. Results were shown as means ± SEM (*n* = 6). ^∗^*p* < 0.05 versus control group; ^#^*p* < 0.05 versus DSS group.

The challenge with DSS in mice damaged colon tissue as shown in HE straining morphology, including crypt loss and leukocyte infiltration; however, these damage signs were attenuated by Gln and Ala-Gln. DSS challenge increased the colon histological index. Interestingly, this increase was significantly ameliorated by Gln and Ala-Gln (Figure 2(a)). In addition, DSS treatment enhanced colon apoptosis as shown in TUNEL-positive nuclei; however, this increase was significantly counteracted by Gln and Ala-Gln (Figure 2(b)). These results indicated that Gln and Ala-Gln restored the DSS-induced colon damage.

### 3.3. Effects of Ala-Gln on Inflammatory Mediators and Immunoglobulins

**Figure 3:**
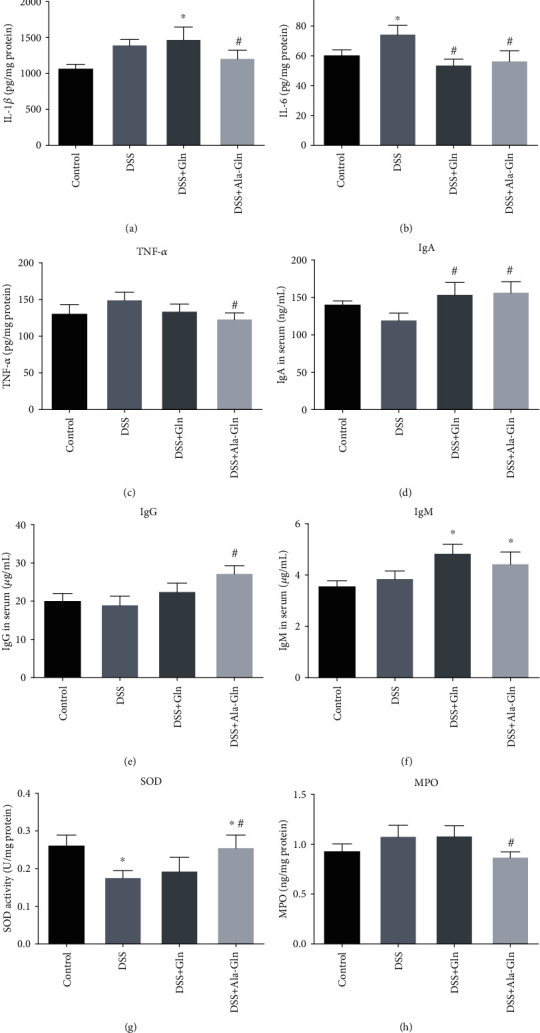
Effect of the supplementations of Gln and Ala-Gln on cytokines, immunoglobulins, SOD, and MPO of mice. (a–c) Concentrations of IL-1*β*, IL-6, and TNF-*α* in colon tissue. (d–f) Concentrations of IgA, IgG, and IgM in serum. (g, h) Contents of SOD and MPO in colon tissue. Results were shown as means ± SEM (*n* = 7). ^∗^*p* < 0.05 versus control group; ^#^*p* < 0.05 versus DSS group.

The contents of inflammatory mediators IL-1*β*, IL-6, and TNF-*α* in colon tissue of DSS-challenged mice were significantly reduced by Gln or Ala-Gln supplementation (*p* < 0.05) (Figures 3(a)–3(c)). The concentrations of immunoglobulins IgA, IgG, and IgM in serum were significantly increased by Ala-Gln (*p* < 0.05) (Figures 3(d)–3(f)). In mouse colon tissues, SOD activity was significantly increased and MPO concentration was significantly decreased by Ala-Gln administration (*p* < 0.05) (Figures 3(g) and 3(h)). These results indicated that Gln and Ala-Gln rescued the immunity status of mice. However, IL-1*β*, TNF-*α*, IgG, SOD, and MPO were not significantly affected by Gln, indicating that Gln is not so effective as Ala-Gln to suppress DSS-induced colitis.

### 3.4. Western Blot

**Figure 4:**
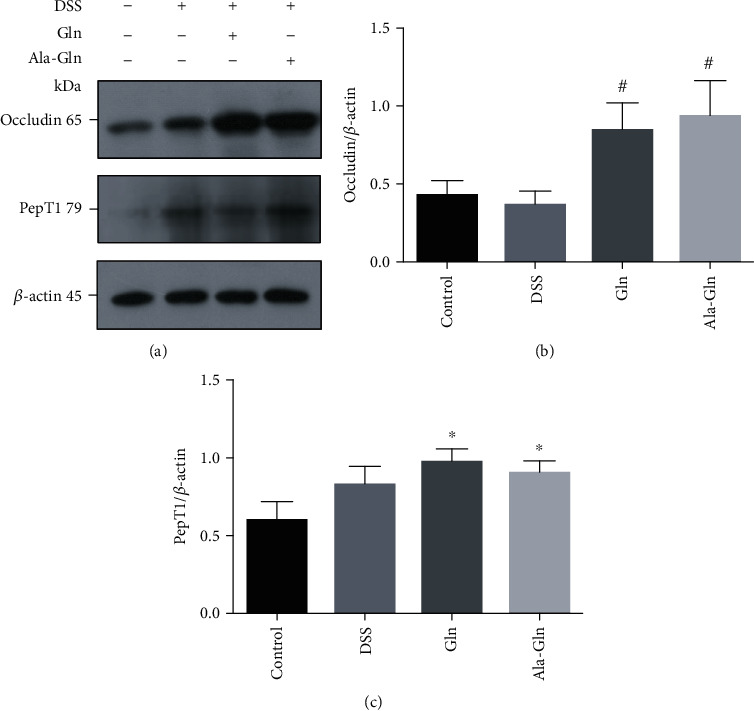
Supplementations of Gln and Ala-Gln increased the protein expressions of occludin and peptide transporter 1 (PepT1) in mouse colon tissue. (a) Representative protein bands. (b, c) Statistical analysis of protein bands of occludin and PepT1. Results were shown as means ± SEM (*n* = 7). ^∗^*p* < 0.05 versus control group; ^#^*p* < 0.05 versus DSS group.

The expression of the tight junction (TJ) protein occludin was significantly increased by Gln and Ala-Gln (*p* < 0.05) (Figures 4(a) and 4(b)), indicating that the intestinal barrier was enhanced by Gln and Ala-Gln to alleviate the colitis. The expression of the peptide transporter PepT1 (Figures 4(a) and 4(c)) was also significantly upregulated by Gln and Ala-Gln (*p* < 0.05), indicating that PepT1 was involved in colitis alleviation by Ala-Gln. However, no significant difference was detected in the expression of phosphorylation MAPK signaling (p38, JNK, and ERK) (data not shown), suggesting that the MAPK signaling pathway was not involved in alleviating intestinal inflammation of Ala-Gln.

### 3.5. Gut Microbiota

**Figure 5:**
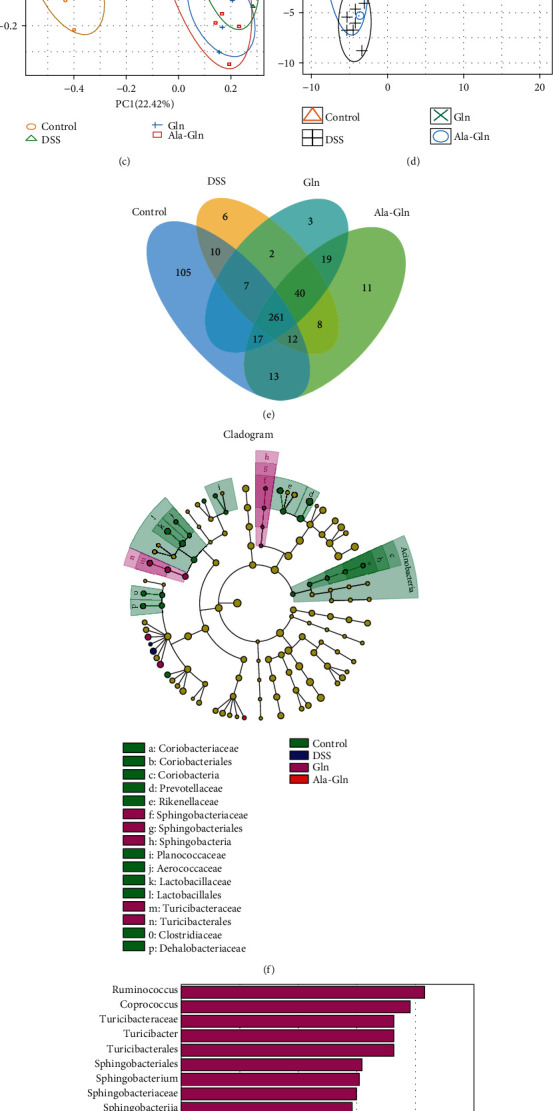
Supplementations of Gln and Ala-Gln altered the colon microbiota in colitic mice. (a, b) Alpha diversity was estimated by using Shannon and Simpson indices. (c, d) Principal component analysis (PCA) plot and partial least squares discriminant analysis (PLS-DA) analysis of gut microbiota. (e) Venn graph for OTUs. (f) LEfSe taxonomic cladogram. Different colors represent biomarker taxon enrichment of control (green), DSS (blue), Gln (purple), and Ala-Gln (red) groups. (g) Linear discriminant analysis (LDA) score (log10) of the different bacterial abundances. Results were shown as means ± SEM (*n* = 7). ^∗^*p* < 0.05, ^∗∗^*p* < 0.01.

The colon microbial community of mice was analyzed by the 16S rDNA phylogenetic method with OTU similarity higher than 97%. Alpha diversity of the microbial communities was estimated by using Shannon and Simpson indices. The Shannon index was significantly decreased by DSS treatment (*p* < 0.01), while the Simpson index was significantly increased by DSS (*p* < 0.05), indicating that DSS decreased the diversity of gut microbial community of mice (Figures 5(a) and 5(b)). Moreover, this decrease was drastically rescued by both Gln and Ala-Gln. The microbiota structure was analyzed by PCA, which showed that the gut microbiota was separated by DSS, Gln, and Ala-Gln treatments (Figures 5(c) and 5(d)). Compared with the DSS group, gut microbiota was separated by Gln and Ala-Gln treatments using OTU-based partial least squares discriminant analysis (PLS-DA) (Figure 5(d)). In addition, the Venn diagram illustrated that 261 universal OTUs were detected out of 521 total OTUs in all samples. There were 105, 6, 3, and 11 unique OTUs in control (total 432), DSS (total 346), Gln (total 356), and Ala-Gln (total 381) groups, respectively (Figure 5(e)). The key bacterial alterations in the taxonomic cladogram showed that *Sphingobacteriaceae*, *Sphingobacteriales*, *Sphingobacteria*, *Turicibacteraceae*, and *Turicibacterales* were the unique cluster markers in the Gln group (Figure 5(f)). The LDA score of taxon abundance illustrated that DSS treatment significantly increased the abundances of *Dorea* and *Defluviitalea*, while Gln significantly increased the abundances of *Ruminococcus* and *Coprococcus* (*p* < 0.05), and Ala-Gln significantly increased *p_75_a5* abundance (*p* < 0.05). Notably, compared with the control group, DSS challenge in all other groups significantly decreased the abundance of probiotics, such as *Prevotellaceae*, *Lactobacillales*, *Lactobacillaceae*, and *Lactobacillus* (*p* < 0.05, Figure 5(g)).

**Figure 6:**
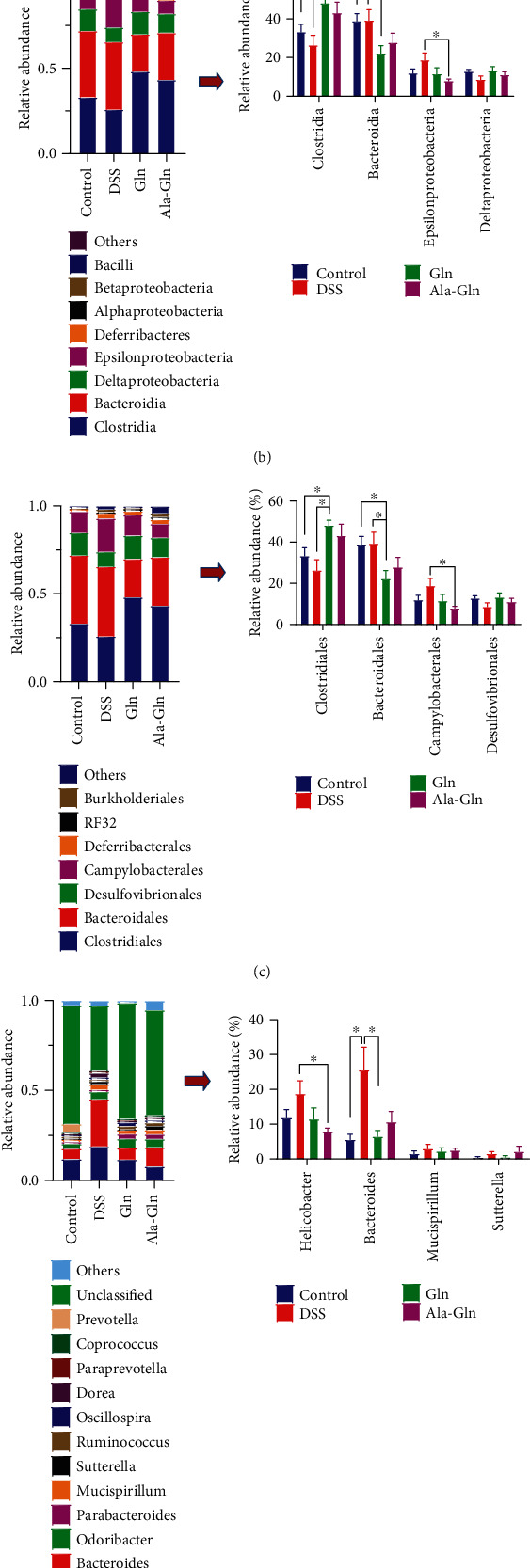
The relative abundances of predominant different bacteria after Gln and Ala-Gln treatments were shown at the phylum (a), class (b), order (c), genus (d), and species (e) levels. Results were shown as means ± SEM (*n* = 7). ^∗^*p* < 0.05; ^∗∗^*p* < 0.01.

The *Firmicutes* and *Bacteroidetes* were the predominant bacteria at the phylum level, and *Clostridia* and *Bacteroidia* were the predominant bacteria at the class level, while *Clostridia* and *Bacteroidales* were the predominant bacteria at the order level (Figures 6(a)–6(c)). At the phylum level, *Bacteroidetes* abundance and the ratio of *Bacteroidetes*/*Firmicutes* were significantly decreased by Gln and Ala-Gln (*p* < 0.05, Figure 6(a)). At the class level, *Clostridia* abundance was significantly increased by Gln, while the abundances of *Bacteroidia* and *Epsilonproteobacteria* were significantly decreased by Gln or Ala-Gln (*p* < 0.05, Figure 6(b)). At the order level, the abundance of *Clostridiales* was significantly increased by Gln, while the abundances of *Bacterioidales* and *Campylobacterales* (gastrointestinal pathogens) were significantly decreased by Gln or Ala-Gln (*p* < 0.05, Figure 6(c)). At the genus level, the abundances of pathogens *Helicobacter* spp. and *Bacteroides* spp. were increased by DSS challenge, while they were significantly decreased by Gln or Ala-Gln to counteract DSS-induced increases (*p* < 0.05, Figure 6(d)). At the species level, the abundance of *Bacteroides_acidifaciens* was significantly increased by DSS challenge, while it was significantly decreased by Gln (*p* < 0.05, Figure 6(e)). These results indicated that the DSS-induced change of gut microbiota was restored by Gln and Ala-Gln.

### 3.6. Effects of Ala-Gln on Metabolism Pathways of Gut Microbial Community

**Figure 7:**
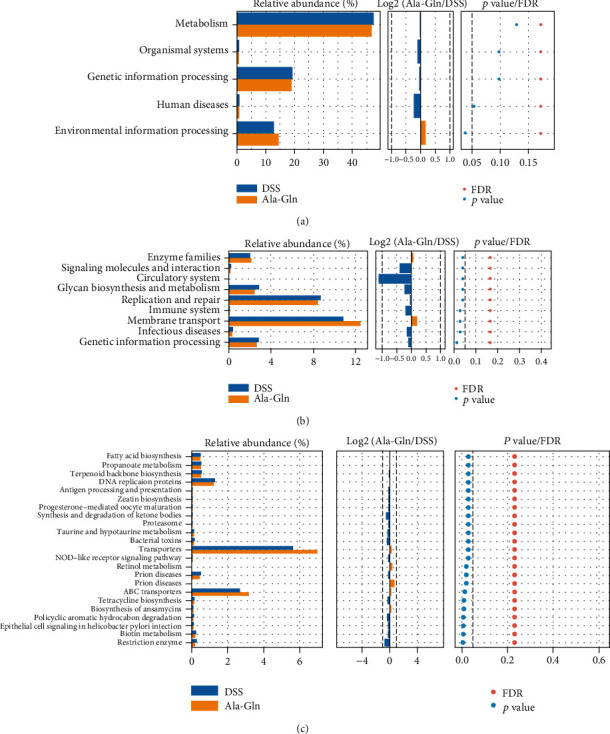
The metabolized pathways of the different microflora abundances after Ala-Gln supplementation in DSS-challenged mice at KEGG levels 1 (a), 2 (b), and 3 (c).

The metabolism of microbial communities was predicted by PICRUSt. At KEGG level 1, the function of environmental information processing was significantly enhanced by Ala-Gln compared with DSS treatment (*p* < 0.05, Figure 7(a)). At KEGG level 2, the functions of membrane transport and enzyme families were significantly enhanced by Ala-Gln (*p* < 0.05, Figure 7(b)). However, the functions of replication and repair, genetic information processing, infectious diseases, immune system, and glycan biosynthesis and metabolism were significantly inhibited by Ala-Gln (*p* < 0.05, Figure 7(b)). At KEGG level 3, compared with the DSS group, the metabolism functions of transporters, ABC transporters, and biosynthesis of ansamycins were significantly enhanced by Ala-Gln treatment. However, the functions of DNA replication proteins, fatty acid biosynthesis, propanoate metabolism, folate biosynthesis, epithelial cell signaling in *Helicobacter pylori* infection, polycyclic aromatic hydrocarbon degradation, restriction enzyme, and bacterial toxins were significantly inhibited by Ala-Gln compared with the DSS treatment (*p* < 0.05, Figure 7(c)).

**Figure 8:**
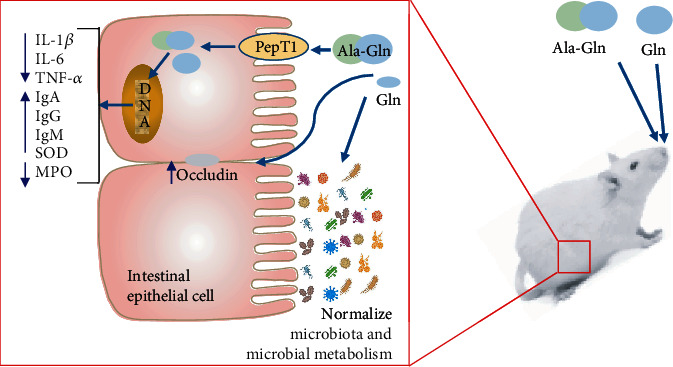
A schematic of the underlying mechanism of Ala-Gln and Gln in alleviating the colitis of mice.

From the above results, the underlying mechanism of Ala-Gln in alleviating colitis of mice can be concluded as follows: (1) the transport of Ala-Gln via PepT1 into intestinal epithelial cells, (2) the decrease in IL-1*β*, IL-6, TNF-*α*, and MPO and the increase in IgA, IgG, IgM, and SOD, and (3) the normalization of microbiota and microbial metabolism (Figure 8).

## 4. Discussion

The IBD is a complex chronic intestinal inflammation associated with the nutrition and gut microbiome [1]. As we know, the use of drugs to manage IBD has many side effects. It was reported that bioactive peptides derived from food proteins had anti-inflammatory function with the potential to treat IBD without side effects [1, 20, 21], such as peptides IRW, IQW [22], EWP [23], GLTSK [24], glycomacropeptide [25], KPV [26], QCQCAVEGGL [27], RILSILRHQNLLKELQDLAL [28], VPY [29], WH [30], P-317 [31], pyroGlu-Leu [32], *γ*-EC, and *γ*-EV [33]. Bioactive peptides can cross the intestinal epithelial wall and enter intact into the bloodstream having bioactive function; therefore, the efficiency to transport amino acids can be enhanced by peptides [20, 34]. In addition, Gln is a therapeutic candidate for improving intestinal health based on its influence on repairing the intestinal epithelial wall and enhancing immunity [35]. Several studies have been performed to investigate the beneficial effects of Gln and Ala-Gln on colitis and intestinal health [3, 9, 15, 36]. However, little is known about the difference between Gln and Ala-Gln in colitis and gut microbiota in the intestinal inflammation. In addition, a dose of 750 mg Ala-Gln/kg BW/d (equal 500 mg Gln/kg BW/d) was used to manage mice based on previous reports of the safe and effective treated dose [9, 10, 15].

The DSS-induced colitic mice are an intensively used model to study IBD with the evaluation of DAI, BW, colon length, signaling pathways, inflammatory cytokines, and gut microbiome in mice [1, 17, 37]. In this study, high DAI, short colon length, and colon tissue damage were observed in DSS-challenged mice, indicating that the colitis model was established successfully [38]. However, these IBD markers were restored by the oral supplement of Ala-Gln, indicating that the mouse colitis was attenuated by Ala-Gln. As shown in this study, it has been confirmed that Ala-Gln attenuated colon inflammation and increased colon length [9]. The cytokines IL-1*β*, IL-6, IL-8, and TNF-*α* can cause intestinal inflammation [1], and their release can induce intestinal dysfunction [37]. The intestinal inflammatory cytokine disorders always are associated with oxidative stress imbalance [39, 40]. In this study, the decreases in the contents of IL-1*β*, IL-6, and TNF-*α* caused by Ala-Gln were confirmed by a previous study which showed that Gln reduced the expression of these cytokines in mice with *Pasteurella multocida* vaccine [41]. These results can be explained partly by Gln bioactivity, which can inhibit DSS-induced colitis via the decrease in proinflammatory cytokines and SOD [36]. The Gln has also been found to inhibit inflammatory response by regulating NF-*κ*B activation, which can cause proinflammatory cytokine release [42], while Gln can inhibit inflammatory cytokines and is beneficial for intestinal mucosa in endotoxemic rats [43]. In the current study, the decrease in inflammatory cytokines by Ala-Gln (not by Gln with IL-1*β* and TNF-*α*) indicated that the extent of colitis was ameliorated by Ala-Gln, which has better beneficial effects than Gln. Additionally, the contents of IgA, IgG, and IgM were increased by Ala-Gln to enhance the immune system. It was reported that Gln can increase IL-6 and SIgA concentrations and protect against *Escherichia coli* infection via intestinal innate immunity in mice [2, 44]. These results indicated that dietary Ala-Gln can ameliorate colitis via enhancing immunoglobulin levels.

The TJ barrier is important to intestinal health by modulating gut permeability and IBD pathogenesis [45], and the impairment of its integrity is a crucial reason to cause colitis. The Gln had defensive effects on the maintenance of intestinal barrier integrity in Caco-2 cells [46]. In the present study, the expression of the TJ protein occludin in the colon was upregulated by Ala-Gln. Another reason for the better suppression effect of Ala-Gln than Gln is that Ala-Gln is the substrate of intestinal PepT1, which can transport Ala-Gln efficiently into the blood circulation [20]. Although PepT1 expression can be upregulated in the IBD colon due to its transport of bacterial peptides into cells, PepT1 can also transport Ala-Gln into intestinal cells and bloodstream to inhibit inflammation [47], such as anti-inflammatory peptides VPY [29], KPV [26], IPAV [48], and *β*-Ala-His [49]. Therefore, PepT1 is a promising potential target for managing colitis; however, its mechanism underlying the inhibition of colitis by bioactive peptides still needs further investigation. However, in this study, no significant effect of Ala-Gln was observed on MAPK phosphorylation, indicating that MAPK signaling is not the mechanism for Ala-Gln to ameliorate colitis. In addition, oxidative stress is another mechanism that causes IBD [50], and the SOD activity can be decreased by DSS challenge [16, 22]. As an indicator for colitis extent, MPO can induce tissue damage due to the release of oxyradicals from leukocytes [3]. In this study, Ala-Gln showed defensive effects by increasing SOD, demonstrating that Ala-Gln could inhibit colitis through mediating oxidation resistance. The MPO concentration was increased by DSS and reversed by Ala-Gln, indicating that Ala-Gln has alleviated colitis inflammation.

The microbiota plays a critical role in maintaining gut oxidative environment and altering chronic inflammation of human and animals [51]. In the current study, intestinal microbial composition was changed and the microbial diversity was reduced in colitic mice. Gut microbial diversity was decreased by DSS challenge, while it was significantly increased by Gln and Ala-Gln, indicating that Gln and Ala-Gln reversed DSS-induced decrease in microbial diversity. In this study, the DSS challenge decreased the OTU numbers, while Ala-Gln treatment increased OTUs to counteract their shifts, indicating that the richness of the gut microbiome was normalized by Ala-Gln to a certain extent. It was reported that the increase in *Bacteroidetes* and the ratio of *Bacteroidetes*/*Firmicutes* were the indicators of IBD [17, 37]. In the current study, the microbiota composition was reversed and the ratio of *Bacteroidetes*/*Firmicutes* was decreased by Ala-Gln treatment. It was also observed that Gln increased the *Firmicutes*/*Bacteroidetes* ratio, decreased the abundance of *Firmicutes*, and beneficially changed the bacterial community and activated the innate immunity in the intestine [52]. It was reported that the decrease in *Bacteroides* and the increase in *Firmicutes* have a defensive effect on IBD [53]. The abundance of *Firmicutes* in DSS-induced colitic mice was also increased by serine [37]. The decrease in *Bacteroides* abundance linked to intestinal inflammation can explain the colitis amelioration by Ala-Gln [17]. In our study, Gln increased *Clostridiales* abundance, which can promote the development of intestinal IgA-producing cells and benefits the immunological responses [54]. The Ala-Gln or Gln restored DSS-induced change of *Lactobacillus*, *Bacteroidales*, *Firmicutes*, *Bacteroides_acidifaciens*, *Clostridia*, *Helicobacter*, and *Bacteroides*. These results indicated that Ala-Gln alleviated the DSS-induced decrease in microbial diversity, evenness, and composition and rescued gut microbiota dysbiosis, which was confirmed by a previous study that Gly-Gln reduced the inflammatory response in piglets by modulating gut microbial community and metabolism [5]. The reason may be that Gln can regulate amino acid utilization via intestinal microorganisms [6].

The metabolism functions of gut microbiota were also inhibited by Ala-Gln, such as epithelial cell signaling in *Helicobacter pylori* infection, infectious diseases, polycyclic aromatic hydrocarbon degradation, and bacterial toxins. Among them, *Helicobacter pylori* infection is a key factor in intestinal disease etiology [55]. However, the metabolism of membrane transporters was enhanced by Ala-Gln, indicating that nutritional transporters (e.g., PepT1) may be enhanced to transport more nutrients into epithelial cells and bloodstream to resist inflammation. Furthermore, the role of gut microbiota mediated by Ala-Gln is still lacking; therefore, the modulating mechanism of Ala-Gln on gut microbiota needs to be investigated in the future.

## 5. Conclusion

This study reveals that the oral administration of Gln and Ala-Gln can protect against DSS-induced colitis through alleviating mucosa damage and inflammatory responses, upregulating the expression of TJ and PepT1 proteins and modulating gut microbiota in mice. The DSS-induced shifts of the microbiome (community diversity, evenness, richness, and composition) in the mouse colon were restored by Gln and Ala-Gln supplementation, including *Lactobacillus*, *Bacteroides_acidifaciens*, *Bacteroidales*, *Firmicutes*, *Clostridia*, *Helicobacter*, and *Bacteroides*. The metabolism functions of gut microflora were also rescued by Ala-Gln, including membrane transporters (PepT1), fatty acid and propanoate metabolism, infectious diseases, immune system, and bacterial toxins. Interestingly, Ala-Gln has a better beneficial effect than Gln in alleviating colitis. In conclusion, Ala-Gln can prevent IBD through PepT1, enhancing the intestinal barrier and modulating gut microbiota and their metabolites. This study provides a new insight into the biological function and protective effect of Ala-Gln in managing IBD. Furthermore, the role and the underlying mechanism of PepT1 and gut microbiota in alleviating colitis need to be investigated in the future.

## Data Availability

All data are available upon request.

## Abbreviations

Ala-Gln: Alanyl-glutamine
BW: Body weight
DAI: Disease activity index
DSS: Dextran sulfate sodium
ELISA: Enzyme-linked immunosorbent assay
ERK: Extracellular signal-regulated kinase
Gln: Glutamine
HE: Hematoxylin and eosin
IBD: Inflammatory bowel disease
IgA: Immunoglobulin A
IL-6: Interleukin-6
JNK: c-Jun N-terminal kinase
KEGG: Kyoto Encyclopedia of Genes and Genomes
LDA: Linear discriminant analysis
LEfSe: Linear discriminant analysis effect size
MPO: Myeloperoxidase
PCA: Principal component analysis
PCR: Polymerase chain reaction
PepT1: Peptide transporter 1
PLS-DA: Partial least squares discriminant analysis
PICRUSt: Phylogenetic Investigation of Communities by Reconstruction of Unobserved States
QIIME: Quantitative Insights into Microbial Ecology
SEM: Standard error of the mean
SOD: Superoxide dismutase
TNF-*α*: Tumor necrosis factor-*α*
TUNEL: Terminal deoxynucleotidyl transferase dUTP nick end labeling.

## References

[1] Zhu W., Ren L., Zhang L., Qiao Q., Farooq M. Z., Xu Q. (2020). The potential of food protein-derived bioactive peptides against chronic intestinal inflammation. *Mediators of Inflammation*.

[2] Liu G., Ren W., Fang J. (2017). l-Glutamine and l-arginine protect against enterotoxigenic Escherichia coli infection via intestinal innate immunity in mice. *Amino Acids*.

[3] Chu C.-C., Hou Y.-C., Pai M.-H., Chao C.-J., Yeh S.-L. (2012). Pretreatment with alanyl-glutamine suppresses T-helper-cell-associated cytokine expression and reduces inflammatory responses in mice with acute DSS- induced colitis. *The Journal of Nutritional Biochemistry*.

[4] Hu X., Deng J., Yu T. (2019). ATF4 deficiency promotes intestinal inflammation in mice by reducing uptake of glutamine and expression of antimicrobial peptides. *Gastroenterology*.

[5] Yan Y., Xu B., Yin B. (2020). Modulation of gut microbial community and metabolism by dietary glycyl-glutamine supplementation may favor weaning transition in piglets. *Frontiers in Microbiology*.

[6] Dai Z.-L., Li X.-L., Xi P.-B., Zhang J., Wu G., Zhu W.-Y. (2013). l-Glutamine regulates amino acid utilization by intestinal bacteria. *Amino Acids*.

[7] Satoh J., Tsujikawa T., Fujiyama Y., Bamba T. (2003). Nutritional benefits of enteral alanyl-glutamine supplementation on rat small intestinal damage induced by cyclophosphamide. *Journal of Gastroenterology and Hepatology*.

[8] Tan B., Liu H., He G. (2017). Alanyl-glutamine but not glycyl-glutamine improved the proliferation of enterocytes as glutamine substitution in vitro. *Amino Acids*.

[9] Hou Y.-C., Liu J.-J., Pai M.-H., Tsou S.-S., Yeh S.-L. (2013). Alanyl-glutamine administration suppresses Th17 and reduces inflammatory reaction in dextran sulfate sodium-induced acute colitis. *International Immunopharmacology*.

[10] Hou Y.-C., Pai M.-H., Liu J.-J., Yeh S.-L. (2013). Alanyl-glutamine resolves lipopolysaccharide-induced lung injury in mice by modulating the polarization of regulatory T cells and T helper 17 cells. *The Journal of Nutritional Biochemistry*.

[11] Liu S.-K., Ma L.-B., Yuan Y. (2020). Alanylglutamine relieved asthma symptoms by regulating gut microbiota and the derived metabolites in mice. *Oxidative Medicine and Cellular Longevity*.

[12] Bunpo P., Murray B., Cundiff J., Brizius E., Aldrich C. J., Anthony T. G. (2008). Alanyl-glutamine consumption modifies the suppressive effect of l-asparaginase on lymphocyte populations in mice. *The Journal of Nutrition*.

[13] Araújo C. V., Lazzarotto C. R., Aquino C. C. (2015). Alanyl-glutamine attenuates 5-fluorouracil-induced intestinal mucositis in apolipoprotein E-deficient mice. *Brazilian Journal of Medical and Biological Research*.

[14] Zhou Y., Zhang P., Deng G., Liu X., Lu D. (2012). Improvements of immune status, intestinal integrity and gain performance in the early-weaned calves parenterally supplemented with l-alanyl-l-glutamine dipeptide. *Veterinary Immunology and Immunopathology*.

[15] Hou Y.-C., Chu C.-C., Ko T.-L., Yeh C.-L., Yeh S.-L. (2013). Effects of alanyl-glutamine dipeptide on the expression of colon-inflammatory mediators during the recovery phase of colitis induced by dextran sulfate sodium. *European Journal of Nutrition*.

[16] Wang K., Jin X., Li Q. (2018). Propolis from different geographic origins decreases intestinal inflammation andBacteroidesspp. populations in a model of DSS-induced colitis. *Molecular Nutrition & Food Research*.

[17] Wang K., Jin X., You M. (2017). Dietary propolis ameliorates dextran sulfate sodium-induced colitis and modulates the gut microbiota in rats fed a western diet. *Nutrients*.

[18] Kitajima S., Takuma S., Morimoto M. (2000). Histological analysis of murine colitis induced by dextran sulfate sodium of different molecular weights. *Experimental Animals*.

[19] Zha A., Yuan D., Cui Z. (2020). The evaluation of the antioxidant and intestinal protective effects of baicalin-copper in deoxynivalenol-challenged piglets. *Oxidative Medicine and Cellular Longevity*.

[20] Xu Q., Hong H., Wu J., Yan X. (2019). Bioavailability of bioactive peptides derived from food proteins across the intestinal epithelial membrane: a review. *Trends in Food Science & Technology*.

[21] Zhang H., Hu C. A. A., Kovacs-Nolan J., Mine Y. (2015). Bioactive dietary peptides and amino acids in inflammatory bowel disease. *Amino Acids*.

[22] Liu G., Yan W., Ding S. (2018). Effects of IRW and IQW on oxidative stress and gut microbiota in dextran sodium sulfate-induced colitis. *Cellular Physiology and Biochemistry*.

[23] Lee M., Kovacs-Nolan J., Archbold T. (2009). Therapeutic potential of hen egg white peptides for the treatment of intestinal inflammation. *Journal of Functional Foods*.

[24] Luna-Vital D. A., González de Mejía E., Loarca-Piña G. (2017). Dietary peptides from phaseolus vulgaris L. reduced AOM/DSS-induced colitis-associated colon carcinogenesis in Balb/c mice. *Plant Foods for Human Nutrition*.

[25] Ortega-González M., Capitán-Cañadas F., Requena P. (2014). Validation of bovine glycomacropeptide as an intestinal anti-inflammatory nutraceutical in the lymphocyte-transfer model of colitis. *British Journal of Nutrition*.

[26] Dalmasso G., Charrier-Hisamuddin L., Nguyen H. T. T., Yan Y., Sitaraman S., Merlin D. (2008). PepT1-Mediated Tripeptide KPV Uptake Reduces Intestinal Inflammation. *Gastroenterology.*.

[27] Hwang J.-W., Lee S.-J., Kim Y.-S. (2012). Purification and characterization of a novel peptide with inhibitory effects on colitis induced mice by dextran sulfate sodium from enzymatic hydrolysates of _Crassostrea gigas_. *Fish & Shellfish Immunology*.

[28] Eissa N., Hussein H., Kermarrec L. (2017). Chromofungin ameliorates the progression of colitis by regulating alternatively activated macrophages. *Frontiers in Immunology*.

[29] Kovacs-Nolan J., Zhang H., Ibuki M. (2012). The PepT1-transportable soy tripeptide VPY reduces intestinal inflammation. *Biochimica et Biophysica Acta (BBA) - General Subjects*.

[30] Kobayashi Y., Kovacs-Nolan J., Matsui T., Mine Y. (2015). The anti-atherosclerotic dipeptide, Trp-His, reduces intestinal inflammation through the blockade of L-Type Ca2+ channels. *Journal of Agricultural and Food Chemistry*.

[31] Sobczak M., Zakrzewski P. K., Cygankiewicz A. I. (2014). Anti-inflammatory action of a novel orally available peptide 317 in mouse models of inflammatory bowel diseases. *Pharmacological Reports*.

[32] Wada S., Sato K., Ohta R. (2013). Ingestion of low dose pyroglutamyl leucine improves dextran sulfate sodium-induced colitis and intestinal microbiota in mice. *Journal of Agricultural and Food Chemistry*.

[33] Zhang H., Kovacs-Nolan J., Kodera T., Eto Y., Mine Y. (2015). *γ*-Glutamyl cysteine and *γ*-glutamyl valine inhibit TNF-*α* signaling in intestinal epithelial cells and reduce inflammation in a mouse model of colitis *via* allosteric activation of the calcium-sensing receptor. *Biochimica et Biophysica Acta (BBA) - Molecular Basis of Disease*.

[34] Xu Q., Yan X., Zhang Y., Wu J. (2019). Current understanding of transport and bioavailability of bioactive peptides derived from dairy proteins: a review. *International Journal of Food Science & Technology*.

[35] Kretzmann N. A., Fillmann H., Mauriz J. L. (2008). Effects of glutamine on proinflammatory gene expression and activation of nuclear factor kappa B and signal transducers and activators of transcription in TNBS-induced colitis. *Inflammatory Bowel Diseases*.

[36] Ren W., Yin J., Wu M. (2014). Serum amino acids profile and the beneficial effects of l-arginine or l-glutamine supplementation in dextran sulfate sodium colitis. *PLoS One*.

[37] Zhang H., Hua R., Zhang B., Zhang X., Yang H., Zhou X. (2018). Serine alleviates dextran sulfate sodium-induced colitis and regulates the gut microbiota in mice. *Frontiers in Microbiology*.

[38] Hu L., Wu C., Zhang Z. (2019). Pinocembrin protects against dextran sulfate sodium-induced rats colitis by ameliorating inflammation, improving barrier function and modulating gut microbiota. *Frontiers in Physiology*.

[39] Liao P., Li Y., Li M. (2020). Baicalin alleviates deoxynivalenol-induced intestinal inflammation and oxidative stress damage by inhibiting NF- _*κ*_ B and increasing mTOR signaling pathways in piglets. *Food and Chemical Toxicology*.

[40] Zha A., Cui Z., Qi M. (2020). Dietary baicalin zinc supplementation alleviates oxidative stress and enhances nutrition absorption in deoxynivalenol challenged pigs. *Current Drug Metabolism*.

[41] Chen S., Liu S., Zhang F. (2014). Effects of dietary l-glutamine supplementation on specific and general defense responses in mice immunized with inactivated Pasteurella multocida vaccine. *Amino Acids*.

[42] Fillmann H., Kretzmann N. A., San-Miguel B. (2007). Glutamine inhibits over-expression of pro-inflammatory genes and down- regulates the nuclear factor kappaB pathway in an experimental model of colitis in the rat. *Toxicology*.

[43] Zhou X., Wu X., Yin Y., Zhang C., He L. (2012). Preventive oral supplementation with glutamine and arginine has beneficial effects on the intestinal mucosa and inflammatory cytokines in endotoxemic rats. *Amino Acids*.

[44] Wu M., Xiao H., Liu G. (2016). Glutamine promotes intestinal SIgA secretion through intestinal microbiota and IL-13. *Molecular Nutrition & Food Research*.

[45] Azuma T., Shigeshiro M., Kodama M., Tanabe S., Suzuki T. (2013). Supplemental naringenin prevents intestinal barrier defects and inflammation in colitic mice. *The Journal of Nutrition*.

[46] Beutheu S., Ghouzali I., Galas L., Déchelotte P., Coëffier M. (2013). Glutamine and arginine improve permeability and tight junction protein expression in methotrexate-treated Caco-2 cells. *Clinical Nutrition*.

[47] Ingersoll S. A., Ayyadurai S., Charania M. A., Laroui H., Yan Y., Merlin D. (2012). The role and pathophysiological relevance of membrane transporter PepT1 in intestinal inflammation and inflammatory bowel disease. *American Journal of Physiology-Gastrointestinal and Liver Physiology*.

[48] Oyama M., Van Hung T., Yoda K., He F., Suzuki T. (2017). A novel whey tetrapeptide IPAV reduces interleukin-8 production induced by TNF-*α* in human intestinal Caco-2 cells. *Journal of Functional Foods*.

[49] Son D. O., Satsu H., Kiso Y., Totsuka M., Shimizu M. (2008). Inhibitory effect of carnosine on interleukin-8 production in intestinal epithelial cells through translational regulation. *Cytokine*.

[50] Li B., Alli R., Vogel P., Geiger T. L. (2014). IL-10 modulates DSS-induced colitis through a macrophage-ROS-NO axis. *Mucosal Immunology*.

[51] Ni J., Wu G. D., Albenberg L., Tomov V. T. (2017). Gut microbiota and IBD: causation or correlation?. *Nature Reviews Gastroenterology & Hepatology*.

[52] Ren W., Duan J., Yin J. (2014). Dietary l-glutamine supplementation modulates microbial community and activates innate immunity in the mouse intestine. *Amino Acids*.

[53] Zhang Y., Tan L., Li C., Wu H., Ran D., Zhang Z. (2020). Sulforaphane alter the microbiota and mitigate colitis severity on mice ulcerative colitis induced by DSS. *AMB Express*.

[54] Umesaki Y., Setoyama H., Matsumoto S., Imaoka A., Itoh K. (1999). Differential roles of segmented filamentous bacteria and clostridia in development of the intestinal immune system. *Infection and Immunity*.

[55] Kusters J. G., van Vliet A. H. M., Kuipers E. J. (2006). Pathogenesis of Helicobacter pylori infection. *Clinical Microbiology Reviews*.

